# Theoretical Insights on the High Pressure Behavior of Pentazolate Anion Complex [Co(H_2_O)_4_(N_5_)_2_]·4H_2_O

**DOI:** 10.1038/s41598-019-52232-3

**Published:** 2019-10-30

**Authors:** Guozheng Zhao, Huili Li, Jianfeng Jia, Haishun Wu, Ming Lu

**Affiliations:** 1 Key Laboratory of Magnetic Molecules & Magnetic Information Materials Ministry of Education, The School of Chemistry and Material Science, Shanxi Normal University, Linfen, 041004 P.R. China; 2 School of Chemical Engineering, Nanjing University of Science and Technology, Nanjing, 210094 P.R. China

**Keywords:** Chemistry, Materials science

## Abstract

Periodic dispersion corrected density functional theory (DFT) calculations were carried out to examine the Hirshfeld surface, two dimensional (2D) fingerprint plots, crystal structure, molecular structure and density of state of all-nitrogen pentazolate anion complex [Co(H_2_O)_4_(N_5_)_2_]·4H_2_O under hydrostatic pressure from 0 to 20 GPa. The GGA/PW91-OBS method was applied in the present study. The intercontacts in [Co(H_2_O)_4_(N_5_)_2_]·4H_2_O were analyzed by Hirshfeld surfaces and 2D fingerprint plots. With ascending pressure, the lattice constants, compression rates, bond lengths, bond angles, and density of states change irregularly. Under 11.5, 13.0 and 15.8 GPa, covalent interaction competition is obvious between Co−N and Co−O bonds. It is possible to achieve orderly modification and regulation of the internal structure of [Co(H_2_O)_4_(N_5_)_2_]·4H_2_O by applied pressure. This is in accordance with the results from density of states analysis. The external compression causes the nonuniformity of electron density and the differential covalent interaction between pentazolate anion, coordinated water and atom Co. It is of great significance to interpret inter/intramolecular interaction and structural stability of [Co(H_2_O)_4_(N_5_)_2_]·4H_2_O and provide theoretical guidance for the design of metal complexes of all-nitrogen pentazolate anion.

## Introduction

Polynitrogen compounds have attracted wide attention in the course of pursuing ultra-high energy density materials due to their superior explosive properties and environmentally friendly characteristics^1–3^. However, because of the immensely exothermic formation of triple bond N≡N, polynitrogen compounds satisfy the demand in virtue of the positive heats of formation and extremely large energy release^4,5^. Recently, pentazolate anion (*cyclo*-N_5_^−^), composed of all nitrogen atoms on a planar five membered ring, has been the focus of research in polynitrogen compounds^6^. As a consequence, the investigation of all-nitrogen species poses arduous challenge, but crucial significance in national defense and economy.

In recent years, great breakthroughs have been made in the preparation of *cyclo*-N_5_^−^. In tetrahydrofuran solution, *cyclo*-N_5_^−^ was formed and detected by Bazanov *et al*.^7^. Zhang *et al*. performed the synthesis and characterization of *cyclo*-N_5_^−^, which was generated by the oxidative cleavage of the C–N bond in 3,5-dimethyl-4-hydroxyphenyl pentazole and stabilized as a component in a (N_5_)_6_(H_3_O)_3_(NH_4_)_4_Cl salt^6^. Lu *et al*. reported six metal complexes of *cyclo*-N_5_^−^ [Na(H_2_O)(N_5_)]·2H_2_O, [M(H_2_O)_4_(N_5_)_2_]·4H_2_O (M = Mn, Fe, Co, and Zn) and [Mg(H_2_O)_6_(N_5_)_2_]·4H_2_O^8,9^, which display acceptable thermal stability (decomposition temperatures onset > 100°C) except the Co complex. Under high pressure, metal salts of *cyclo*-N_5_^−^ CsN_5_ and LiN_5_ were synthesized by Oleynik *et al*.^10^ and Laniel *et al*.^11^, respectively. The *cyclo*-N_5_^−^ is stabilized by covalent/ionic bond and hydrogen bond with metal cation and water, respectively. This is consistent with the results that metals can stabilize *cyclo*-N_5_^−^ obtained by Lein *et al*.^12,13^. Aside from metal salts of *cyclo*-N_5_^−^, metal inorganic frameworks (MIFs) of *cyclo*-N_5_^−^, were successfully constructed, such as [Na_8_(N_5_)_8_(H_2_O)_3_]_n_^14^, [Ba(N_5_)(NO_3_)(H_2_O)_3_]_n_^15^, [NaBa_3_(N_5_)_6_(NO_3_)(H_2_O)_3_]_n_^15^, [Cu(N_5_)(N_3_)]_n_^15^, [Ag(N_5_)]_n_^15^, MPF-1^16^, [LiNa(N_5_)_2_(H_2_O)_4_]·H_2_O^17^ and [Ag(NH_3_)_2_]^+^[Ag_3_(N_5_)_4_]^−^^18^, indicating that each nitrogen of *cyclo*-N_5_^−^ can coordinate with different metal atoms in various coordination modes.

Despite several studies have reported the synthesis and characterization of *cyclo*-N_5_^−^ complexes, the underlying and practical problems of metal complexes of *cyclo*-N_5_^−^ are still unclear due to the complex chemical behavior. For example, various behavioral studies have not yet been involved under different pressures. It is crucial to investigate the internal structure within a range of pressures, particularly under high pressure. The reason is that the shockwave with large velocity can produce up to 50 GPa of pressure during detonation process^19,20^. Explosives undergo phase transition and decomposition under these extreme conditions. Therefore, the investigation of metal complexes of *cyclo*-N_5_^−^ under high pressure is significant for the understanding of inter/intramolecular interaction and structural stability. In addition, it also provides better theoretical guidance and early paving for the design and development of metal complexes of *cyclo*-N_5_^−^.

It is a challenging work to study the microscopic properties of metal complexes of *cyclo*-N_5_^−^. Compared with the experimental work, theoretical calculation is a powerful tool to analyze inter/intramolecular interaction and structural stability in physical and chemical fields under high pressure^21,22^. The effect of high pressure on the geometrical and electronic structures of cocrystal NTO/TZTN has been explored by us under hydrostatic pressure of 0–80 GPa^21^. Based on the application of density functional theory (DFT) method with pseudopotentials and a planewave of basis set, the investigation of the relationship between structure and stability was favorably achieved under high pressures. In this study, under the external pressures of 0−20 GPa, the periodic DFT investigation of pressure effect was performed on Hirshfeld surface, two dimensional (2D) fingerprint plots, density of state, crystal and molecular structures of [Co(H_2_O)_4_(N_5_)_2_]·4H_2_O.

## Results and Discussion

### Hirshfeld surface and 2D fingerprint plots

Hirshfeld surface analysis and 2D fingerprint plots describe the types and regions of intercontacts in crystal stacking, which can explore explicit inter/intramolecular atom–atom close contacts and provide a measurable value to the fraction of a special close contact^23,24^. The structure a from experimental crystal [Co(H_2_O)_4_(N_5_)_2_]·4H_2_O serves as the input file. The relative contributions to Hirshfeld surface for the close contacts are illustrated by Fig. 2. It is obvious that the fractions of H···H (22.0%), H···N (27.2%), O···O (15.1%), N···H (13.9%), and O···H (9.2%) contacts are more than those of other contacts. The H···N, N···H, and O···H contacts display the spike features in Fig. 2, considering to hydrogen bond interactions^23^, which play an important role in the stability of the whole system. The shortest distance of H···N and O···H are 3.00 Å and 2.15 Å, belonged to weak and medium hydrogen bond, respectively^23,25^. The O··O contact is not conducive to the stability of system, which lead to the increasing repulsion. Therefore, for crystal design, avoiding or reducing O··O contact is helpful to reduce the total energy of system.

**Figure 1:**
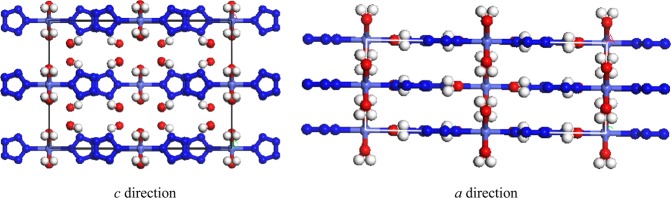
Perspective views of [Co(H_2_O)_4_(N_5_)_2_]·4H_2_O along *a* and *c* directions. (Materials Studio 6.0, https://www.3dsbiovia.com/products/collaborative-science/biovia-materials-studio/).

**Figure 2:**
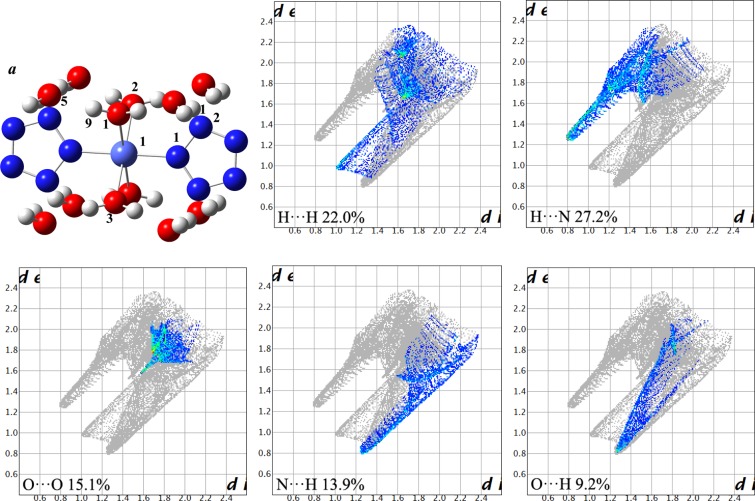
The structure a and relative contributions to Hirshfeld surface for the close contacts in experimental crystal stacking. Light blue, red, blue and white balls represent Co, O, N and H atoms, respectively. The full fingerprint is marked by gray shadow. (GaussView 5.0, http://gaussian.com/ and Crystal Explorer 3.1, http://crystalexplorer.scb.uwa.edu.au/index.html).

**Figure 3:**
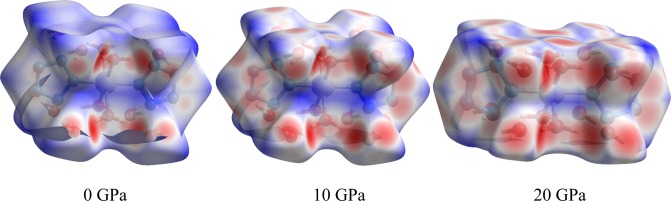
Hirshfeld surfaces mapped with *d*_norm_ in [Co(H_2_O)_4_(N_5_)_2_]·4H_2_O under 0, 10, and 20 GPa. (Crystal Explorer 3.1, http://crystalexplorer.scb.uwa.edu.au/index.html).

Figure 3 exhibits the Hirshfeld surfaces of structure a under 0, 10, and 20 GPa. At 0 GPa, the blue regions predominate on Hirshfeld surfaces. As the pressure increases to 20 GPa, the blue area decreases and the red area increases, which indicated that the relative contributions to Hirshfeld surface for the closer contacts increase with ascending pressure. Actually, with ascending pressure, the molecules are getting closer and closer to achieve the reduction of structural spaces, increasing the intermolecular interactions, which demonstrate that a theoretical insight to investigate the structural response is obtained by Hirshfeld surface analysis under an applied pressure.

**Figure 4:**
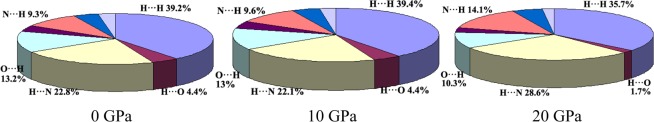
The relative contributions of close contacts to Hirshfeld surface under 0, 10, and 20 GPa.

To elaborate information on the Hirshfeld surface in detail, the corresponding contributions of close contacts under 0, 10, and 20 GPa are pictured in Fig. 4. The relative contributions of H···N and N···H contacts increase from 22.8% to 28.6% and from 9.3% to 14.1%, respectively, under the whole pressures. In contrast, the contributions of H···O and O···H contacts decrease to 1.7% and 10.3%, respectively. These observations indicate that hydrogen bonds are the main interactions between N, O and H atoms. The existence of hydrogen bonds O−H···N ensure the planarity and conjugation of pentazolate anion rings. In addition, the hydrogen bonds O−H···O further achieve the maximum bonding between constitution water and coordinated water to ensure the minimum energy, which is in line with the results from experimental results^8^.

External pressure is essential to the formation of hydrogen bond interaction. From the above analysis, it can be seen that pressure is more conducive to the formation of O−H···N hydrogen bonds between pentazolate anion and constitution water. The reason is that the larger space, the easier compress, which match well with the reduction of blue area on Hirshfeld surfaces. Another obvious change is that O···O contacts are almost negligible under external pressure, thus avoiding space repulsion to a large extent. At 0 GPa, the H···N and O···H contacts are 2.26 and 1.78 Å, respectively, while those are 1.87 and 1.51 Å, respectively, at 20 GPa indicating that the hydrogen bonds shorten, but the O···H contacts is less compressible than H···N contacts under the pressure ranging from 0 to 20 GPa.

### Crystal and molecular structure

**Figure 5:**
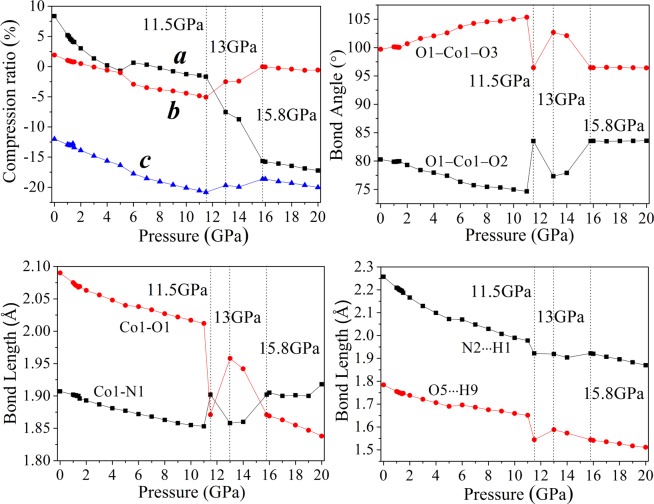
The description of compression ratios, bond angles and bond lengths of [Co(H_2_O)_4_(N_5_)_2_]·4H_2_O under various pressures.

The transformation of molecular conformation and the formation of denser packed materials can be induced by external pressure due to the variable conformation of molecule. To obtain insight into high pressure behavior of [Co(H_2_O)_4_(N_5_)_2_]·4H_2_O, the crystal structure was analyzed in the pressure range of 0−20 GPa. With the ascending pressure, the lattice constants (*a*, *b*, *c*) show irregular change. The compression ratio as a function of pressure is described in Fig. 5. Under hydrostatic pressure from 0 to 20 GPa, the irregular changes are also reflected in the variation of compression ratio. According to Fig. 5, the compression rates *a*, *b* and *c* basically reduce except the pressure region between 11.5 and 15.8 GPa, which is abnormal, compared with other pressure ranges. It is indicated that [Co(H_2_O)_4_(N_5_)_2_]·4H_2_O may undergo noticeable structural changes or transformations in the pressure range from 11.5 to 15.8 GPa. The compression ratios along *a*, *b* and *c* directions are not identical in a sequence of *c* > *a* > *b*. Under 20 GPa, the compression ratios along *a*, *b* and *c* directions are 17.23%, 0.57% and 20.02%, respectively, meaning that the compressibility of [Co(H_2_O)_4_(N_5_)_2_]·4H_2_O is anisotropic and the structure in *a* direction is stiffer than *b* and *c* directions. The bulk modulus of [Co(H_2_O)_4_(N_5_)_2_]·4H_2_O is 16.7 GPa, which is higher than that of rare gas^26^, and lower those of MOFs^27^ and gold(I) iodide^28^. The bulk modulus and fracture strength increase under hydrostatic pressure from 0 to 20 GPa, which the reason is that the fracture strength is proportional to the bulk modulus, while the compressibility of [Co(H_2_O)_4_(N_5_)_2_]·4H_2_O decrease. With the structural changes or transformations of [Co(H_2_O)_4_(N_5_)_2_]·4H_2_O are taken place between 11.5 and 15.8 GPa, the investigation of molecular structure is followed.

The selected bond lengths and angles of [Co(H_2_O)_4_(N_5_)_2_]·4H_2_O under various pressures are plotted in Fig. 5. Covalent interaction competition was found in [Co(H_2_O)_4_(N_5_)_2_]·4H_2_O under compression, which is exhibited by Fig. 5. The competition between Co−N and Co−O bonds was examined with different pressures. When the pressure increases to 20 GPa, the structural space is condensed and the covalent and hydrogen bonds are changed in geometric structure. From Fig. 5, before 11 GPa, Co−N and Co−O bonds shorten constantly, meanwhile, the covalent interactions enhance correspondingly. Under hydrostatic pressure from 11.0 to 11.5 GPa, the lengths of bonds Co1−N1 and Co1−O1 increase 0.049 Å (2.64%) and decrease 0.141 Å (7.01%), respectively. The change of other Co−N and Co−O bonds is in line with those of Co1−N1 and Co1−O1, respectively. In addition, the bond angles of O1−Co1−O2 and O1−Co1−O3 increase 8.88° and decrease 8.88°, respectively. The coordinated waters approach the Co atoms and the pentazolate anions are far away from the Co atoms, which is shown that the Co−O covalent interaction predominates in the competition between Co−N and Co−O bonds. Compared to 11.5 GPa, at 13.0 GPa, the bond lengths of Co1−N1 and Co1−O1 decrease 0.044 Å (2.31%) and increase 0.087 Å (4.65%), respectively. The covalent interaction of Co−N is stronger than that of Co−O. The bond angles of O1−Co1−O2 and O1−Co1−O3 decrease 6.21° and increase 6.21°, respectively. With the pressure increasing from 14.0 GPa to 15.8 GPa, it can be seen that the bond lengths of Co1−N1 and Co1−O1 increase 0.042 Å (2.26%) and decrease 0.087 Å (4.65%), respectively. Correspondingly, the bond angles of O1−Co1−O2 and O1−Co1−O3 increase 5.64° and decrease 5.64°, respectively. It is suggested that the Co−O covalent interaction predominates in the competition between Co−N and Co−O bonds again. Pressure plays an important role in the covalent interaction competition between Co−N and Co−O bonds. It is possible to achieve orderly modification and regulation of the internal structure of [Co(H_2_O)_4_(N_5_)_2_]·4H_2_O by applied pressure. Furthermore, from 11.0 GPa to 15.8 GPa, it is opposite for the change trend of Co1−O1 bond length and O1−Co1−O2 bond angle. The shorter the bond length of Co1−O1, the bigger the bond angle of O1−Co1−O2. The reason is that as the bond length of Co1−O1 decreases, the distance between two oxygen atoms becomes closer and closer, and the repulsion increases. Besides the covalent interaction competition between Co−N and Co−O bonds, the repulsion between oxygen atoms also has a great influence on the stability of system. In the whole pressure range, the pentazolate anion rings and Co atoms maintain favorable planarity and conjugation, which contribute greatly to the stability of system, but with the increase of pressure, steric hindrance increases. The hydrogen bond interactions of N2···H1 and O5···H9 increase generally from 0 to 20 GPa. The change of other N···H and O···H hydrogen bonds is consistent with those of N2···H1 and O5···H9, respectively. This is mainly due to the increasing closeness between pentazolate anion, coordinated water and constitution water under external pressure.

### Density of state

**Figure 6:**
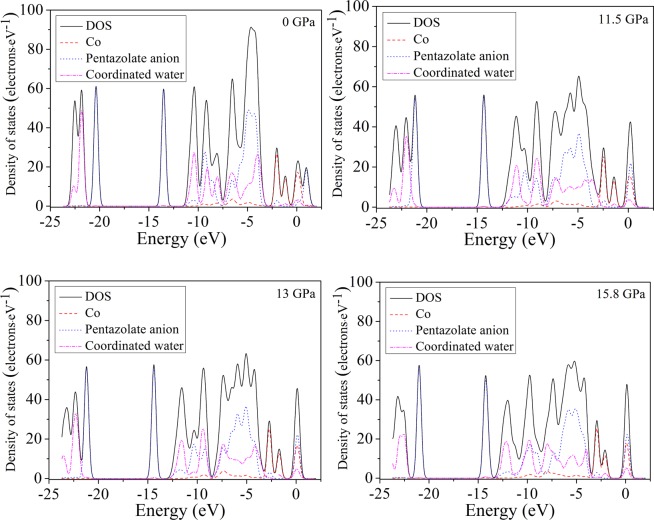
Calculated total DOS and PDOS for Co atoms, pentazolate anion and coordinated water under different pressures.

The total density of state (DOS) and partial density of state (PDOS) were investigated for a detailed explain on the electronic structure of [Co(H_2_O)_4_(N_5_)_2_]·4H_2_O under various pressures^29^. Figure 6 displays the calculated total DOS and PDOS for Co atoms, pentazolate anion and coordinated water. From Fig. 6, the higher the pressure, the wider peak of DOS curve, which illustrates that the electrons move freely between valence and conduction bands. The crystal compression causes the better mobility of electrons and stronger delocalization of system. Under the applied pressure, the conduction bands shift to the lower energy, which reduces the band gap and increases the possibility of electron excitation. From −25 to −2.5 eV, the DOS is mainly composed of pentazolate anion and coordinated water. In contrast, from −2.5 to 2.5 eV, Co atom and pentazolate anion contribute to the DOS. It is indicated that the delocalization of pentazolate anion results in the appearance of electrons in the whole energy bands.

From 11.0 to 11.5 GPa, at the Fermi energy level, the PDOS peaks of pentazolate anion and coordinated water increase 0.017 and 0.790 electrons·eV^−1^, respectively, meaning that the change of electron density between atoms Co and O is higher than that between atoms Co and N. This is consistent with the result that the Co−O covalent interaction predominates in the competition between Co−N and Co−O bonds. When the pressure reaches 13.0 GPa, at the Fermi energy level, it is noticeable that the PDOS peaks of pentazolate anion and coordinated water increase 0.246 and 0.124 electrons·eV^−1^, respectively. The electron density between atoms Co and N varies greatly. Under hydrostatic pressure from 13.0 to 15.8 GPa, at the Fermi energy level, the PDOS peaks of pentazolate anion and coordinated water increase 0.156 and 0.655 electrons·eV^−1^, respectively, which prove that Co−O covalent interaction is dominant in the competition between Co−N and Co−O bonds. These conclusions correspond well with the analysis from the molecular structure section. Under hydrostatic pressure, the nonuniform distribution of electron density leads to the change of interactions between pentazolate anion, coordinated water and atom Co. Due to the external pressure, the electron densities on pentazolate anion and coordinated water change, which causes the change of the lengths of bonds Co−N and Co−O. The electron density nonuniformity of [Co(H_2_O)_4_(N_5_)_2_]·4H_2_O plays an irreplaceable role in the covalent interaction competition between Co−N and Co−O bonds.

## Conclusions

Pressure-induced Hirshfeld surface, 2D fingerprint plots, crystal structure, molecular structure and density of state of pentazolate anion complex [Co(H_2_O)_4_(N_5_)_2_]·4H_2_O were investigated by periodic density functional theory calculations in the pressure range of 0–20 GPa. The GGA with OBS correction to PW91 (PW91-OBS) functional provides more reliable lattice parameters than the others. For experimental structure **a**, the contact fractions of H···N (27.2%), N···H (13.9%), O···H (9.2%), and O···O (15.1%) show the competition between hydrogen bond interaction and steric repulsion. Under various pressure, the existence of hydrogen bonds O−H···N ensure the planarity and conjugation of pentazolate anion ring and hydrogen bonds O−H···O achieve the maximum hydrogen bonding between constitution water and coordinated water. Between 11.5 and 15.8 GPa, the abnormal compression rates reveal that [Co(H_2_O)_4_(N_5_)_2_]·4H_2_O undergo distinct structural changes or transformations due to the covalent interaction competition between Co−N and Co−O bonds, which match well with the analysis from bond lengths, bond angles, and density of states under the hydrostatic pressure of 11.5, 13.0, and 15.8 GPa. The electron density nonuniformity of [Co(H_2_O)_4_(N_5_)_2_]·4H_2_O was caused by external pressure, which induces differential covalent interaction between pentazolate anion, coordinated water and atom Co and the change of lengths of Co−N and Co−O bonds.

### Computational details

The first-principle computations were carried out based on the application of density functional theory (DFT) method with the Vanderbilt-type ultrasoft pseudopotential^30,31^ implemented in Materials Studio’s CASTEP module^32,33^. The cutoff energy of plane waves and *k*-point grid were set to 500 eV and 2 × 2 × 3, respectively. The total energy, residual force, displacement of atoms, and residual bulk stress were converged less than 1.0 × 10^−5^ eV, 0.03 eV·Å^−1^, 0.001 Å, and 0.05 GPa, respectively. The experimental crystal was taken from Lu *et al*. (CCDC 1527750)^8^. The [Co(H_2_O)_4_(N_5_)_2_]·4H_2_O crystallize in orthorhombic space group *Fmmm* (Fig. 1).

Hirshfeld surface and two dimensional (2D) fingerprint plots were presented and analyzed by Crystal Explorer 3.1 program^34^. The normalized contact distance (*d*_norm_) mapped on Hirshfeld surface is shown by Eq. (1):

1 $${d}_{norm}=\frac{{d}_{i}-{r}_{i}^{vdw}}{{r}_{i}^{vdw}}+\frac{{d}_{e}-{r}_{e}^{vdw}}{{r}_{e}^{vdw}}$$

where *d*_i_ and *d*_e_ mean the distances from the surface to the nearest atom inside and outside the surface, respectively; ${r}_{i}^{vdw}$ and ${r}_{e}^{vdw}$ mean the van der Waals (vdW) radius of two atoms inside and outside the surface, respectively. The *d*_norm_ was mapped on Hirshfeld surface with colors range from −0.461 Å (red) to 1.526 Å (blue). The 2D fingerprint plots were generated through Hirshfeld surfaces, which displayed the dimensional summary of intermolecular interactions for the close contacts.

## Supplementary information

Supplementary information

## Data Availability

The data that support the findings of this study are available from the corresponding authors on request.
