# Supplementary material for: Theoretical Insights on the High Pressure Behavior of Pentazolate Anion Complex [Co(H2O)4(N5)2]·4H2O
Source: Sci Rep. 2019 Oct 30;9:15648. doi: 10.1038/s41598-019-52232-3 (PMC6821737; doi:10.1038/s41598-019-52232-3)
Supplement: Supplementary file 1 — Supplementary information [file 41598_2019_52232_MOESM1_ESM.doc]

**Supplementary information**

**1. Computational methods**

The first-principle calculations were performed by the application of density functional theory (DFT) method with the combination of Vanderbilt-type ultrasoft pseudopotential and a plane-wave expansion of the wave functions [1] implemented in the CASTEP [2] module of Materials Studio 6.0 [3]. The self-consistent ground state of system was determined by using a band-by-band conjugated gradient technique to minimize the total energy of the system with respect to the plane-wave coefficients. The electronic wave functions were obtained in a density-mixing minimization method [4] for the self-consistent field calculation and the structures were relaxed using the Broyden, Fletcher, Goldfarb, and Shannon (BFGS) [5] method. The cutoff energy of plane waves was set to 500 eV. Brillouin zones sampling was carried out by using the Monkhost−Pack scheme with a *k*-point grid of 2 × 2 × 3. The values of the kinetic energy cutoff and the *k*-point grid were determined to ensure the convergence of total energies.

The initial crystal was taken from Lu *et al*. (CCDC 1527750) [6] and designed to the following computations. All the calculations are on the basis of the same experimental crystal structure of [Co(H_2_O)_4_(N_5_)_2_]·4H_2_O. The total energy of the system was converged less than 1.0 × 10^-5^ eV, the residual force less than 0.03 eV⋅Å^-1^, the displacement of atoms less than 0.001 Å, and the residual bulk stress less than 0.05 GPa.

**2. Functionals selection**

**Table 1** Comparison between relaxed lattice parameters of [Co(H_2_O)_4_(N_5_)_2_]·4H_2_O and experimental data at ambient pressure *^a^*

| Method | *a* (Å) | *b* (Å) | *c* (Å) | Vol. (Å^3^) |
| --- | --- | --- | --- | --- |
| GGA/PBE | 19.427 (0.60) | 18.226 (0.06) | 6.097 (-0.06) | 2158.6 (0.61) |
| GGA/PBE-TS | 13.524 (0.11) | 17.952 (0.05) | 6.004 (-0.07) | 1457.5 (0.08) |
| GGA/PBE-G06 | 13.807 (0.14) | 17.850 (0.04) | 5.941 (-0.08) | 1464.1 (0.09) |
| GGA/PW91 | 14.861 (0.22) | 18.284 (0.07) | 6.172 (-0.05) | 1677.0 (0.25) |
| GGA/PW91-OBS | 13.199 (0.09) | 17.545 (0.03) | 5.743 (-0.11) | 1330.0 (-0.01) |
| Exp. | 12.144 | 17.114 | 6.465 | 1343.7 |

*^a^* The values in parentheses correspond to the percentage differences relative to the experimental data.

Five different functionals, generalized gradient approximation (GGA), as a test, were applied to the computation of [Co(H_2_O)_4_(N_5_)_2_]·4H_2_O. To benchmark the performance of theoretical approach, GGA/PBE (Perdew-Burke-Ernzerhof) [7], GGA/PW91(Perdew-Wang-91) [8] and dispersion corrections were selected to fully relax the [Co(H_2_O)_4_(N_5_)_2_]·4H_2_O without any constraint at ambient pressure. The experimental and relaxed cell parameters of [Co(H_2_O)_4_(N_5_)_2_]·4H_2_O are listed by Table 1. The relative errors of the calculated values to the experimental ones indicate that the calculated values of GGA/PW91-OBS is much closer to the experimental ones than the others. The GGA/PW91-OBS method is more suitable for studying [Co(H_2_O)_4_(N_5_)_2_]·4H_2_O, which is different from the previous studies in term of energetic crystals [9,10] show that more reliable lattice parameters could be produced by the LDA functional than the GGA. Therefore, GGA/PW91-OBS method has been employed in the present study.

**References**

[1] D. Vanderbilt, [Phys. Rev. B](http://www.baidu.com/link?url=MUJomNUIW0nG4C6aLPWTYZMnrZHtve637zdomKZAP7PkQyd_ZxvCcmy7aPVB2nTn2x4gkH9sShCwiCO4q8TS1a) 41 (1990) 7892.

[2] S.J. [Clark,](https://vpn2.jiangnan.edu.cn/,DanaInfo=apps.webofknowledge.com,SSL+OneClickSearch.do?product=UA&search_mode=OneClickSearch&SID=N23rhMGtjYPmxvO8sma&field=AU&value=Clark, SJ&ut=3631209&pos={2}&excludeEventConfig=ExcludeIfFromFullRecPage) M.D. Segall, C.J. Pickard, P.J. Hasnip, M.J. Probert, K. Refson, M.C. Payne, Z. Krist-Cryst Mater. 220 (2005) 567.

[3] Materials Studio 6.0, Accelrys Inc, San Diego, 2012.

[4] G. Kresse, J. Furthmuller, [Phys. Rev. B](http://www.baidu.com/link?url=MUJomNUIW0nG4C6aLPWTYZMnrZHtve637zdomKZAP7PkQyd_ZxvCcmy7aPVB2nTn2x4gkH9sShCwiCO4q8TS1a) 54 (1996) 11169.

[5] R. Fletcher, in: Practical Methods of Optimization, Wiley, New York, 1980, pp. 101.

[6] Y.G. Xu, Q. Wang, C. Shen, Q.H. Lin, P.C. Wang, M. Lu, Nature 549 (2017) 78.

[7] J.P. Perdew, K. Burke, M. Ernzerhof, [Phys. Rev. Lett.](http://www.baidu.com/link?url=valTirtxbghRib9dGq2UmIjM7lYE27tLI1YJZLuVyTH2jLS8ZL6yrvuCNP2Fcf_C9-ysMiT7HAc9zy67x4kFDq) 77 (1996) 3865.

[8] J.P. Perdew, Y. Wang, [Phys. Rev. B](http://www.baidu.com/link?url=MUJomNUIW0nG4C6aLPWTYZMnrZHtve637zdomKZAP7PkQyd_ZxvCcmy7aPVB2nTn2x4gkH9sShCwiCO4q8TS1a) 45 (1992) 13244.

[9] Z.C. Liu, Q. Wu, W.H. Zhu, H.M. Xiao, RSC Adv. 5 (2015) 34216.

[10] Q. Wu, W.H. Zhu, H.M. Xiao, J. Phys. Chem. C 117 (2013) 16830.
